# Crystal structure of 1,1′-{(dodecane-1,12-di­yl)bis­[(aza­niumylyl­idene)methanylyl­idene]}bis­(naphthalen-2-olate)

**DOI:** 10.1107/S2056989015007938

**Published:** 2015-04-25

**Authors:** Kamel Ouari, Moufida Merzougui, Sabrina Bendia, Corinne Bailly

**Affiliations:** a Laboratoire d’Electrochimie, d’Ingénierie Moléculaire et de Catalyse Redox, Faculty of Technology, University of Ferhat Abbas Sétif, 19000 Sétif, Algeria; b Service de Radiocristallographie, Institut de Chimie de Strasbourg, UMR 7177 CNRS–Unistra, 1 rue Blaise Pascal, Strasbourg 67008, France

**Keywords:** crystal structure, 1,12-di­amino­dodeca­ne, 2-hy­droxy-1-naphthaldehyde, hydrogen bonds

## Abstract

The title compound, C_34_H_40_N_2_O_2_, exists in an extended conformation and has crystallographically imposed centrosymmetry. The crystal packing can be described as being composed of parallel layers stacked along [010]. The zwitterionic structure is stabilized by an intra­molecular N—H⋯O hydrogen-bond inter­action.

## Related literature   

The compound is synthesized using two procedures, the ultrasound and the conventional methods. We found that the ultrasound irradiation method is more convenient and efficient. For conventional synthesis of similar compounds, see: Ouari *et al.* (2015*a* ▸); Mohammadi & Rastegari (2012 ▸); Bhowmik *et al.* (2011 ▸). For ultrasonic synthesis of similar compounds, see: Rayati & Abdolalian (2013 ▸); Khan *et al.* (2014 ▸); Kanagarajan *et al.* (2011 ▸). For related crystal structures, see: Ouari *et al.* (2010 ▸, 2015*b* ▸); Popović *et al.* (2001 ▸); Friscic *et al.* (1998 ▸); Bi *et al.* (2012 ▸); Temel *et al.* (2010 ▸). For their applications, see: Köse *et al.* (2015 ▸); Grivani *et al.* (2013 ▸); Amin *et al.* (2010 ▸); Panneerselvam *et al.* (2009 ▸); Nasr *et al.* (2009 ▸); Nejo *et al.* (2009 ▸); Taha *et al.* (2012 ▸). 
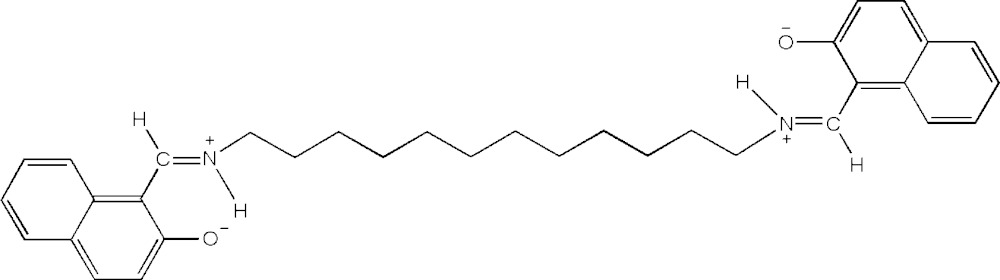


## Experimental   

### Crystal data  

C_34_H_40_N_2_O_2_
*M* *_r_* = 508.68
Monoclinic, 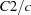

*a* = 54.400 (5) Å
*b* = 4.7465 (4) Å
*c* = 10.7022 (9) Å
β = 96.318 (2)°
*V* = 2746.6 (4) Å^3^
*Z* = 4
Mo *K*α radiation
μ = 0.08 mm^−1^
*T* = 173 K
0.50 × 0.14 × 0.06 mm

### Data collection  

Bruker APEXII CCD diffractometer
Absorption correction: multi-scan (*SADABS*; Bruker, 2008 ▸) *T*_min_ = 0.682, *T*_max_ = 0.746
17506 measured reflections
3271 independent reflections
2313 reflections with *I* > 2σ(*I*)
*R*_int_ = 0.036

### Refinement  

*R*[*F* ^2^ > 2σ(*F* ^2^)] = 0.048
*wR*(*F* ^2^) = 0.125
*S* = 1.04
3271 reflections
176 parameters
H atoms treated by a mixture of independent and constrained refinement
Δρ_max_ = 0.24 e Å^−3^
Δρ_min_ = −0.19 e Å^−3^

### 

Data collection: *APEX2* (Bruker, 2008 ▸); cell refinement: *SAINT* (Bruker, 2008 ▸); data reduction: *SAINT*; program(s) used to solve structure: *SHELXS97* (Sheldrick, 2008 ▸); program(s) used to refine structure: *SHELXL2013* (Sheldrick, 2015 ▸); molecular graphics: *SHELXTL* (Sheldrick, 2008 ▸); software used to prepare material for publication: *SHELXTL*.

## Supplementary Material

Crystal structure: contains datablock(s) I, New_Global_Publ_Block. DOI: 10.1107/S2056989015007938/mw2131sup1.cif

Structure factors: contains datablock(s) I. DOI: 10.1107/S2056989015007938/mw2131Isup2.hkl

**Fig. 1.**
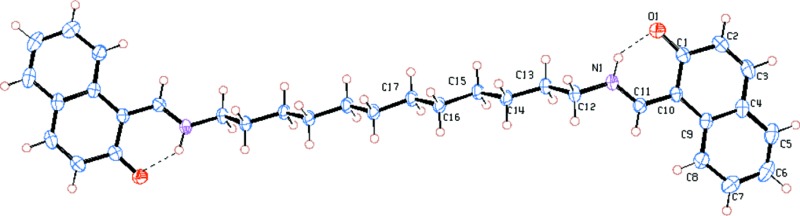
The title compound with atom-labelling scheme. Displacement ellipsoids are drawn at the 50% probability level. H atoms are represented as small spheres of arbitrary radius.

**Fig. 2.**
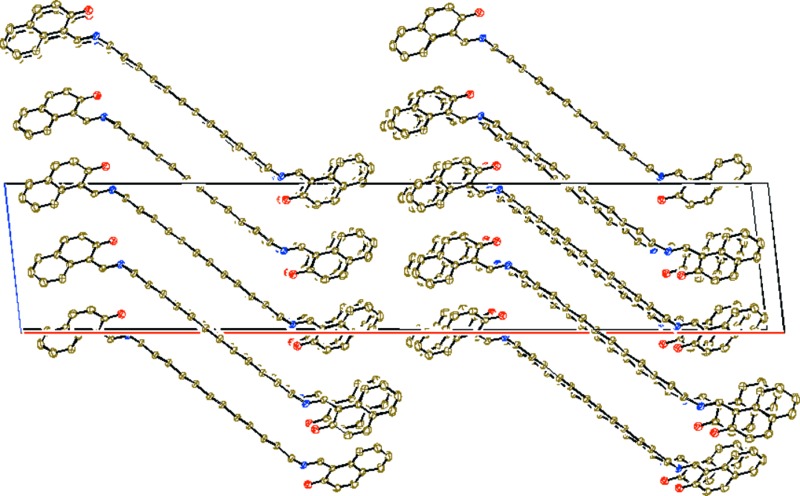
Crystal packing of the title compound viewed along the c axis.

. DOI: 10.1107/S2056989015007938/mw2131fig1.tif
The title compound with atom-labelling scheme. Displacement ellipsoids are drawn at the 50% probability level. H atoms are represented as small spheres of arbitrary radius.

c . DOI: 10.1107/S2056989015007938/mw2131fig2.tif
Crystal packing of the title compound viewed along the *c* axis.

CCDC reference: 1032833

Additional supporting information:  crystallographic information; 3D view; checkCIF report

## Figures and Tables

**Table 1:** Hydrogen-bond geometry (, )

*D*H*A*	*D*H	H*A*	*D* *A*	*D*H*A*
N1H1*N*O1	0.94(2)	1.75(2)	2.5498(18)	140.6(19)

## supplementary crystallographic information

### S0.1. Synthesis and crystallization

Ultrasonication method

A reaction flask containing 0.344g (2mmol) of 2-hy­droxy-1-naphthaldehyde and 0.508g (1mmol) of 1,12-di­amino­dodecane, mixed and ground to a fine powder in a mortar, was immersed in an ultrasonic bath containing water at a temperature of 50 °C. The reaction mixture was exposed to ultrasound irradiation for 40 min. Upon completion, based on TLC analysis (silica gel, CH_2_Cl_2_/MeOH, 9.5/0.5, V/V) the product was washed with methanol (3 x 3 mL) and di­ethyl ether (3 x 3 mL) and filtered. Single crystals, suitable for X-ray diffraction, were obtained after 2 days of crystallization from DMSO/MeOH.

Color: Yellow, Yield: 88 %, mp: 148°C. Analysis calculated for C_34_H_40_N_2_O_2_: C, 80.27; H, 7.92; N, 5.50%; found: C, 80.06; H, 7.80; N, 5.78%.

Conventional method

To a solution of 0.172 g (1mmol) of 2-hy­droxy-1-naphthaldehyde in 5 mL of methanol was added 0.254 g (0.5 mmol) of 1,2-di­amino­dodecane dissolved in 5 mL of the same solvent.The mixture was stirred and refluxed for 3 hours under a nitro­gen atmosphere. At completion, based on TLC analysis, the resulting compound was filtered and washed with methanol and di­ethyl ether to afford pure product in 62% yield.

### S0.2. Refinement

The iminium H atom was located from a difference Fourier map and refined isotropically. C-bound H atoms were included in calculated positions and treated as riding atoms: C—H = 0.95 Å (CH) or 0.99 Å (CH_2_) with Uiso(H) = 1.2Ueq (C—Har.).

### S1. Results and discussion

Schiff base ligands can be easily synthesized using conventional or ultrasonic irradiation methods by reacting primary amines and carbonyl compounds in which the azomethine bond is formed and they can used to form complexes (Ouari *et al.*, 2015a., Mohammadi *et al.*, 2012; Bhowmik *et al.*, 2011., Grivani *et al.*, 2013; Nejo *et al.*, 2009., Rayati *et al.*, 2013., Khan *et al.*, 2014., Kanagarajan *et al.*, 2011).

The synthesis via ultrasound irradiation is an efficient, fast, high yielding method and is a more economical synthetic process for the preparation of the Schiff base compound than the conventional method.

The azomethine group >C=N of the Schiff base can form stable metal complexes by coordinating through the nitro­gen atom (Ouari *et al.*, 2015b., Ouari *et al.*, 2010 ). Schiff base ligands have many applications including anti-microbial agents (Köse *et al.*, 2015., Taha *et al.*;2012., Panneerselvam *et al.*, 2009), anti-tumor agents, (Nasr *et al.*, 2009) and as xanthine oxidase inhibitors (Amin *et al.*, 2010).

This compound crystallized in the monoclinic space group *C*2/*c*, whereas the related compounds(C_26_H_24_N_2_O_2_, C_28_H_28_N_2_O_2_) (Friscic *et al.*, 1998), (C_28_H_26_N_2_O_2_) (Bi *et al.*, 2012) and (C_28_H_20_N_2_O_2_—CHCL_3_) (Popović *et al.*, 2001) crystallized in the orthorhombic space groups Pbca, Pbcn, P2_1_2_1_2_1_, and P2_1_2_1_2_1_, respectively. The hydrogen atom in the title compound is located on the nitro­gen atom (Fig.1). The C1—O1 bond length of 1.2802 (19)Å indicates double-bond character while the N1—C11 bond length of 1.2994 (19)Å indicates single-bond character thus confirming the zwitterionic formulation. Similar results have been reported (Temel *et al.*, 2010]. The crystal packing can be described as parallel chains along the *c* axis (Fig. 2). It is stabilized by intra­molecular N—H···O hydrogen bonding (Table 1) and by weak inter­molecular C—H···π ring inter­actions. These inter­actions link the molecules within the layers and also link the layers together thereby reinforcing the cohesion of the ionic structure.

### Crystal data

**Table:**

C_34_H_40_N_2_O_2_	*F*(000) = 1096
*M**_r_* = 508.68	*D*_x_ = 1.230 Mg m^−^^3^
Monoclinic, *C*2/*c*	Mo *K*α radiation, λ = 0.71073 Å
*a* = 54.400 (5) Å	Cell parameters from 3931 reflections
*b* = 4.7465 (4) Å	θ = 3.0–27.8°
*c* = 10.7022 (9) Å	µ = 0.08 mm^−^^1^
β = 96.318 (2)°	*T* = 173 K
*V* = 2746.6 (4) Å^3^	Prism, yellow
*Z* = 4	0.50 × 0.14 × 0.06 mm

### Data collection

**Table:**

Bruker APEXII CCD diffractometer	3271 independent reflections
Radiation source: sealed tube	2313 reflections with *I* > 2σ(*I*)
Triumph monochromator	*R*_int_ = 0.036
φ and ω scans	θ_max_ = 27.9°, θ_min_ = 2.3°
Absorption correction: multi-scan (*SADABS*; Bruker, 2008)	*h* = −70→70
*T*_min_ = 0.682, *T*_max_ = 0.746	*k* = −6→5
17506 measured reflections	*l* = −14→14

### Refinement

**Table:**

Refinement on *F*^2^	Primary atom site location: structure-invariant direct methods
Least-squares matrix: full	Secondary atom site location: difference Fourier map
*R*[*F*^2^ > 2σ(*F*^2^)] = 0.048	Hydrogen site location: mixed
*wR*(*F*^2^) = 0.125	H atoms treated by a mixture of independent and constrained refinement
*S* = 1.04	*w* = 1/[σ^2^(*F*_o_^2^) + (0.0503*P*)^2^ + 2.2119*P*] where *P* = (*F*_o_^2^ + 2*F*_c_^2^)/3
3271 reflections	(Δ/σ)_max_ < 0.001
176 parameters	Δρ_max_ = 0.24 e Å^−^^3^
0 restraints	Δρ_min_ = −0.19 e Å^−^^3^

### Special details

**Table:**

Geometry. All e.s.d.'s (except the e.s.d. in the dihedral angle between two l.s. planes) are estimated using the full covariance matrix. The cell e.s.d.'s are taken into account individually in the estimation of e.s.d.'s in distances, angles and torsion angles; correlations between e.s.d.'s in cell parameters are only used when they are defined by crystal symmetry. An approximate (isotropic) treatment of cell e.s.d.'s is used for estimating e.s.d.'s involving l.s. planes.

### Fractional atomic coordinates and isotropic or equivalent isotropic displacement parameters (Å^2^)

**Table:**

	*x*	*y*	*z*	*U*_iso_*/*U*_eq_	
C1	0.11107 (3)	−0.3639 (3)	1.08101 (14)	0.0273 (3)	
C2	0.09446 (3)	−0.5389 (3)	1.14296 (15)	0.0345 (4)	
H2	0.1011	−0.6682	1.2056	0.041*	
C3	0.06975 (3)	−0.5229 (4)	1.11383 (17)	0.0386 (4)	
H3	0.0594	−0.6413	1.1570	0.046*	
C4	0.05853 (3)	−0.3340 (4)	1.02027 (16)	0.0329 (4)	
C5	0.03264 (3)	−0.3230 (4)	0.99173 (19)	0.0448 (5)	
H5	0.0225	−0.4413	1.0362	0.054*	
C6	0.02183 (3)	−0.1460 (5)	0.90169 (19)	0.0478 (5)	
H6	0.0043	−0.1410	0.8833	0.057*	
C7	0.03675 (3)	0.0275 (4)	0.83679 (19)	0.0434 (4)	
H7	0.0293	0.1510	0.7737	0.052*	
C8	0.06204 (3)	0.0223 (4)	0.86294 (16)	0.0357 (4)	
H8	0.0718	0.1428	0.8175	0.043*	
C9	0.07384 (3)	−0.1580 (3)	0.95574 (14)	0.0267 (3)	
C10	0.10041 (3)	−0.1712 (3)	0.98713 (13)	0.0248 (3)	
C11	0.11636 (3)	0.0138 (3)	0.93099 (14)	0.0261 (3)	
H11	0.1092	0.1468	0.8714	0.031*	
C12	0.15676 (3)	0.2039 (3)	0.90007 (15)	0.0287 (3)	
H12A	0.1668	0.3092	0.9673	0.034*	
H12B	0.1468	0.3419	0.8467	0.034*	
C13	0.17384 (3)	0.0464 (3)	0.82076 (15)	0.0280 (3)	
H13A	0.1638	−0.0594	0.7537	0.034*	
H13B	0.1838	−0.0912	0.8743	0.034*	
C14	0.19108 (3)	0.2470 (3)	0.76130 (15)	0.0284 (3)	
H14A	0.2013	0.3492	0.8288	0.034*	
H14B	0.1810	0.3879	0.7102	0.034*	
C15	0.20806 (3)	0.0982 (3)	0.67812 (14)	0.0282 (3)	
H15A	0.2186	−0.0370	0.7301	0.034*	
H15B	0.1978	−0.0108	0.6129	0.034*	
C16	0.22456 (3)	0.2977 (3)	0.61412 (14)	0.0289 (3)	
H16A	0.2346	0.4090	0.6793	0.035*	
H16B	0.2140	0.4308	0.5611	0.035*	
C17	0.24187 (3)	0.1500 (3)	0.53257 (14)	0.0292 (3)	
H17A	0.2526	0.0186	0.5858	0.035*	
H17B	0.2319	0.0369	0.4680	0.035*	
N1	0.14027 (2)	0.0119 (3)	0.95644 (12)	0.0288 (3)	
O1	0.13443 (2)	−0.3838 (3)	1.11158 (11)	0.0359 (3)	
H1N	0.1458 (4)	−0.130 (5)	1.014 (2)	0.065 (7)*	

### Atomic displacement parameters (Å^2^)

**Table:**

	*U*^11^	*U*^22^	*U*^33^	*U*^12^	*U*^13^	*U*^23^
C1	0.0341 (8)	0.0276 (7)	0.0212 (7)	−0.0011 (6)	0.0076 (6)	−0.0064 (6)
C2	0.0435 (10)	0.0325 (8)	0.0281 (8)	−0.0035 (7)	0.0066 (7)	0.0023 (7)
C3	0.0423 (10)	0.0389 (9)	0.0367 (9)	−0.0129 (8)	0.0131 (8)	0.0029 (8)
C4	0.0314 (8)	0.0353 (9)	0.0335 (9)	−0.0074 (7)	0.0105 (7)	−0.0075 (7)
C5	0.0312 (9)	0.0536 (11)	0.0510 (12)	−0.0139 (8)	0.0115 (8)	−0.0061 (9)
C6	0.0245 (9)	0.0620 (13)	0.0568 (12)	−0.0024 (8)	0.0033 (8)	−0.0112 (10)
C7	0.0336 (9)	0.0506 (11)	0.0449 (11)	0.0052 (8)	−0.0008 (8)	−0.0043 (9)
C8	0.0300 (9)	0.0396 (9)	0.0377 (9)	−0.0007 (7)	0.0048 (7)	0.0002 (7)
C9	0.0273 (8)	0.0277 (8)	0.0262 (7)	−0.0021 (6)	0.0075 (6)	−0.0076 (6)
C10	0.0271 (8)	0.0262 (7)	0.0221 (7)	−0.0016 (6)	0.0077 (6)	−0.0050 (6)
C11	0.0281 (8)	0.0282 (7)	0.0231 (7)	0.0020 (6)	0.0073 (6)	−0.0030 (6)
C12	0.0267 (8)	0.0326 (8)	0.0286 (8)	−0.0028 (6)	0.0107 (6)	−0.0011 (6)
C13	0.0259 (8)	0.0324 (8)	0.0271 (8)	0.0001 (6)	0.0097 (6)	−0.0008 (6)
C14	0.0233 (7)	0.0346 (8)	0.0286 (8)	0.0003 (6)	0.0087 (6)	−0.0003 (6)
C15	0.0265 (7)	0.0341 (8)	0.0253 (8)	0.0002 (6)	0.0092 (6)	−0.0001 (6)
C16	0.0256 (8)	0.0366 (8)	0.0259 (8)	0.0003 (6)	0.0093 (6)	−0.0023 (6)
C17	0.0270 (8)	0.0354 (8)	0.0267 (8)	0.0002 (6)	0.0096 (6)	−0.0008 (6)
N1	0.0259 (7)	0.0345 (7)	0.0275 (7)	−0.0004 (6)	0.0096 (5)	0.0016 (6)
O1	0.0323 (6)	0.0435 (7)	0.0317 (6)	0.0024 (5)	0.0026 (5)	0.0042 (5)

### Geometric parameters (Å, º)

**Table:**

C1—O1	1.2802 (19)	C12—N1	1.4553 (19)
C1—C10	1.433 (2)	C12—C13	1.522 (2)
C1—C2	1.442 (2)	C12—H12A	0.9900
C2—C3	1.348 (2)	C12—H12B	0.9900
C2—H2	0.9500	C13—C14	1.524 (2)
C3—C4	1.430 (2)	C13—H13A	0.9900
C3—H3	0.9500	C13—H13B	0.9900
C4—C5	1.409 (2)	C14—C15	1.525 (2)
C4—C9	1.413 (2)	C14—H14A	0.9900
C5—C6	1.362 (3)	C14—H14B	0.9900
C5—H5	0.9500	C15—C16	1.518 (2)
C6—C7	1.394 (3)	C15—H15A	0.9900
C6—H6	0.9500	C15—H15B	0.9900
C7—C8	1.374 (2)	C16—C17	1.5232 (19)
C7—H7	0.9500	C16—H16A	0.9900
C8—C9	1.411 (2)	C16—H16B	0.9900
C8—H8	0.9500	C17—C17^i^	1.518 (3)
C9—C10	1.449 (2)	C17—H17A	0.9900
C10—C11	1.414 (2)	C17—H17B	0.9900
C11—N1	1.2994 (19)	N1—H1N	0.94 (2)
C11—H11	0.9500		
			
O1—C1—C10	122.68 (14)	N1—C12—H12B	109.3
O1—C1—C2	119.65 (15)	C13—C12—H12B	109.3
C10—C1—C2	117.67 (14)	H12A—C12—H12B	108.0
C3—C2—C1	121.38 (16)	C12—C13—C14	111.58 (13)
C3—C2—H2	119.3	C12—C13—H13A	109.3
C1—C2—H2	119.3	C14—C13—H13A	109.3
C2—C3—C4	122.38 (15)	C12—C13—H13B	109.3
C2—C3—H3	118.8	C14—C13—H13B	109.3
C4—C3—H3	118.8	H13A—C13—H13B	108.0
C5—C4—C9	120.10 (17)	C13—C14—C15	113.24 (13)
C5—C4—C3	120.99 (16)	C13—C14—H14A	108.9
C9—C4—C3	118.92 (15)	C15—C14—H14A	108.9
C6—C5—C4	121.31 (17)	C13—C14—H14B	108.9
C6—C5—H5	119.3	C15—C14—H14B	108.9
C4—C5—H5	119.3	H14A—C14—H14B	107.7
C5—C6—C7	119.16 (17)	C16—C15—C14	113.62 (13)
C5—C6—H6	120.4	C16—C15—H15A	108.8
C7—C6—H6	120.4	C14—C15—H15A	108.8
C8—C7—C6	120.85 (18)	C16—C15—H15B	108.8
C8—C7—H7	119.6	C14—C15—H15B	108.8
C6—C7—H7	119.6	H15A—C15—H15B	107.7
C7—C8—C9	121.47 (17)	C15—C16—C17	113.89 (13)
C7—C8—H8	119.3	C15—C16—H16A	108.8
C9—C8—H8	119.3	C17—C16—H16A	108.8
C8—C9—C4	117.11 (14)	C15—C16—H16B	108.8
C8—C9—C10	123.66 (14)	C17—C16—H16B	108.8
C4—C9—C10	119.23 (14)	H16A—C16—H16B	107.7
C11—C10—C1	118.31 (14)	C17^i^—C17—C16	113.82 (17)
C11—C10—C9	121.19 (14)	C17^i^—C17—H17A	108.8
C1—C10—C9	120.43 (13)	C16—C17—H17A	108.8
N1—C11—C10	123.61 (15)	C17^i^—C17—H17B	108.8
N1—C11—H11	118.2	C16—C17—H17B	108.8
C10—C11—H11	118.2	H17A—C17—H17B	107.7
N1—C12—C13	111.46 (13)	C11—N1—C12	123.87 (14)
N1—C12—H12A	109.3	C11—N1—H1N	112.7 (13)
C13—C12—H12A	109.3	C12—N1—H1N	123.4 (13)
			
O1—C1—C2—C3	179.64 (16)	C2—C1—C10—C11	176.23 (13)
C10—C1—C2—C3	0.1 (2)	O1—C1—C10—C9	179.73 (14)
C1—C2—C3—C4	0.2 (3)	C2—C1—C10—C9	−0.7 (2)
C2—C3—C4—C5	179.79 (17)	C8—C9—C10—C11	4.3 (2)
C2—C3—C4—C9	0.1 (2)	C4—C9—C10—C11	−175.79 (13)
C9—C4—C5—C6	0.5 (3)	C8—C9—C10—C1	−178.81 (14)
C3—C4—C5—C6	−179.20 (17)	C4—C9—C10—C1	1.1 (2)
C4—C5—C6—C7	−0.1 (3)	C1—C10—C11—N1	2.6 (2)
C5—C6—C7—C8	−0.2 (3)	C9—C10—C11—N1	179.54 (14)
C6—C7—C8—C9	0.1 (3)	N1—C12—C13—C14	179.84 (13)
C7—C8—C9—C4	0.3 (2)	C12—C13—C14—C15	−178.46 (13)
C7—C8—C9—C10	−179.84 (16)	C13—C14—C15—C16	177.58 (14)
C5—C4—C9—C8	−0.5 (2)	C14—C15—C16—C17	179.05 (13)
C3—C4—C9—C8	179.13 (15)	C15—C16—C17—C17^i^	179.32 (17)
C5—C4—C9—C10	179.57 (15)	C10—C11—N1—C12	−179.25 (14)
C3—C4—C9—C10	−0.8 (2)	C13—C12—N1—C11	−116.21 (16)
O1—C1—C10—C11	−3.3 (2)		

Symmetry code: (i) −*x*+1/2, −*y*+1/2, −*z*+1.

### Hydrogen-bond geometry (Å, º)

**Table:**

*D*—H···*A*	*D*—H	H···*A*	*D*···*A*	*D*—H···*A*
N1—H1*N*···O1	0.94 (2)	1.75 (2)	2.5498 (18)	140.6 (19)
