# Supplementary figures and images for: Crystal structure of 1,1′-{(dodecane-1,12-di­yl)bis­[(aza­niumylyl­idene)methanylyl­idene]}bis­(naphthalen-2-olate)
Source: Acta Crystallogr E Crystallogr Commun. 2015 Apr 25;71(Pt 5):o351–2. doi: 10.1107/S2056989015007938 (PMC4420129; doi:10.1107/S2056989015007938)

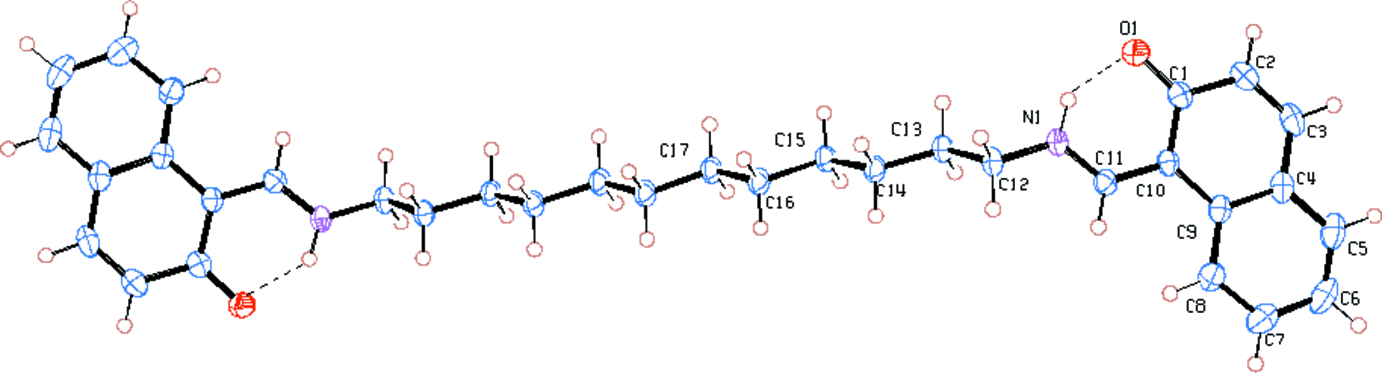

Supplement: Supplementary file 3 [file e-71-0o351-fig1.tif]

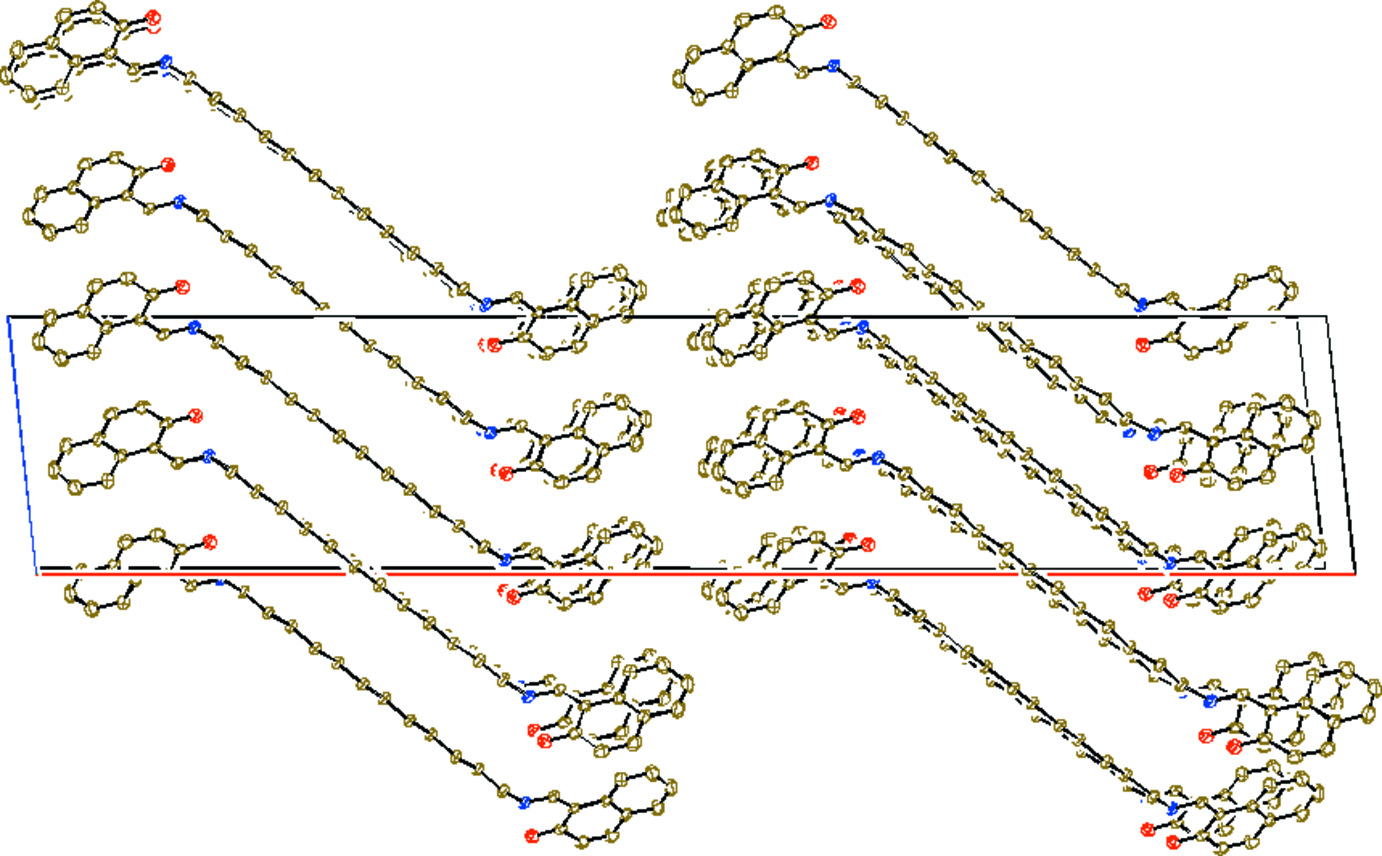

Supplement: Supplementary file 4 [file e-71-0o351-fig2.tif]
